# Innovation in pest management: can the past inform the future?

**DOI:** 10.1002/ps.71202

**Published:** 2026-08-10

**Authors:** Eric Moog, Franck E. Dayan

**Affiliations:** ^1^ Department of Agricultural Biology Colorado State University Fort Collins CO USA

## Abstract

Innovation arises from exploring unknown areas of knowledge. In most instances, progress occurs incrementally, slowly expanding the boundary of what is known. Occasionally, though, transformative discoveries reveal new areas of research that ‘we didn't know that we didn't know.’ With respect to agriculture, the Green Revolution was achieved by cumulative innovation rather than a single breakthrough, though some giant steps were made along the way. Advances in engineering (mechanization), genetics (breeding) and chemistry (fertilizers and pesticides) that enable modern agriculture were built slowly. Looking ahead, artificial intelligence is expected to accelerate agricultural discovery by integrating vast datasets from mechanization, genetics, and chemistry. Autonomous machinery, robotics, drones, and hyperspectral sensing may enable precision pest management with reduced labor and environmental impact. Gene‐editing technologies such as CRISPR could rapidly develop climate‐ and pest‐resilient crops, while emerging non‐chemical approaches, including RNA interference (RNAi) and peptide‐based agents, as well as lasers and electric tools, may provide more targeted crop protection. © 2026 The Author(s). *Pest Management Science* published by John Wiley & Sons Ltd on behalf of Society of Chemical Industry.

## Graphical abstract

Progress in pest management occurred incrementally, but occasionally, transformative discoveries introduced disruptive technologies. Looking ahead, generative artificial intelligence is expected to accelerate agricultural discovery by integrating vast datasets from mechanization, genetics, and chemistry.

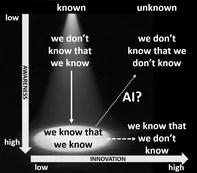

## 1 PERSPECTIVE

A thought‐provoking article by David Shiffman in *American Scientist* questioned ‘Why are we funding this?’ in the context of scientific research and discovery.^1^ This article caused us to reflect on how modern agriculture has come to be what it is today, and to ponder how current research and development will shape the future of agriculture. While it is difficult to predict the future, we know that Plato's proverb ‘Necessity is the mother of invention’ has served humanity well so far. Historically, crises have acted as catalysts for creativity and technological advancement.^2^ While our current agricultural practices face a myriad of difficulties, failure to feed the world is not an option, and new technologies are emerging (and will continue to do so) to address these challenges.^3^, ^4^ The fundamental question is which disruptive technologies will enable a second ‘Green Revolution.’

From a philosophy of logic perspective, the relationship between various states of knowledge with respect to awareness and innovation can be illustrated by a modified ‘Johari Window’ (Fig. 1). In this matrix of ‘known’ and ‘unknown,’ the illuminated surface area represents what ‘we know that we know,’ as the sum of all the knowledge currently available. Research in this area of high awareness leads to low innovation outcome (duplicative research). In instances involving ‘unknown knowns’ where ‘we don't know that we know,’ hidden assets have already been discovered, but their potential use has not been realized for a particular purpose. Exploiting this knowledge does not increase the body of knowledge (illuminated surface area) and is likely to have a low impact innovation.

**Figure 1:**
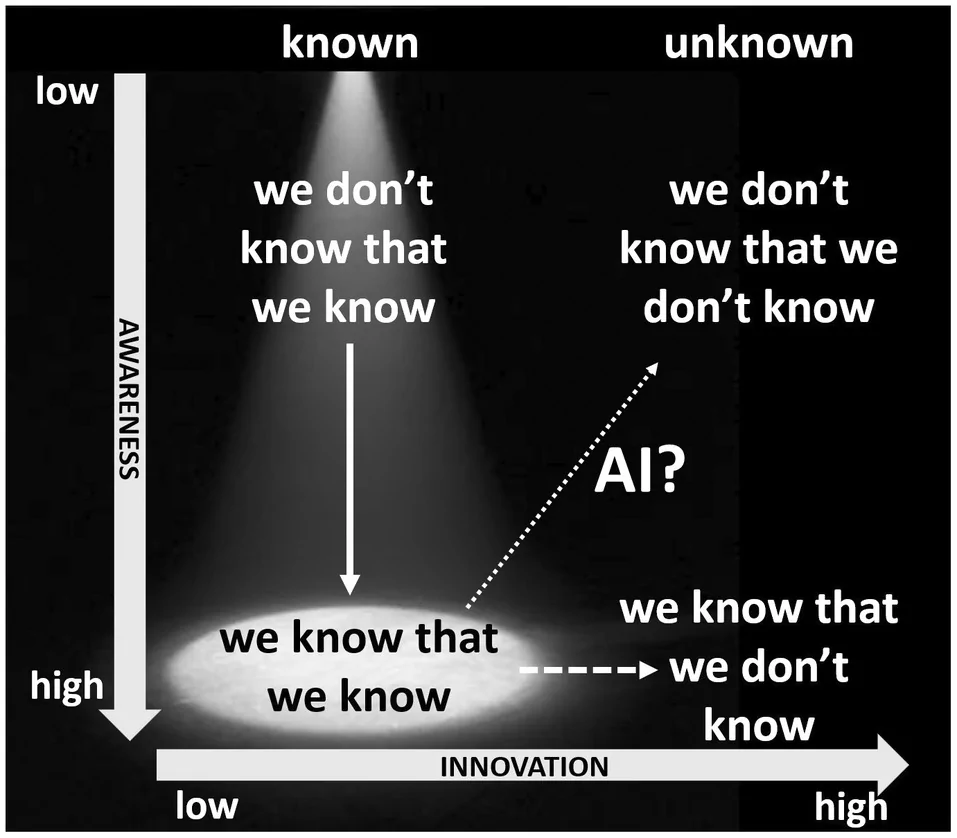
Modified Johari Window matrix illustrating the relationship between various states of knowledge with respect to awareness and innovation. Thin downward arrow represents duplicative research, thin dashed arrow represents increase in knowledge due to traditional research and development programs, thin dotted arrow represents unexpected transformative discoveries that lead to new disruptive technologies that may be facilitated using artificial intelligence (AI).

A more promising route is to increase knowledge by expanding the boundary of the illuminated surface area. In this quadrant, a relatively high awareness informs us that ‘we know that we don't know.’ This is historically how scientific research has led to incremental innovation through testing new research hypotheses based on prior knowledge to explore knowledge gaps. The most inventive approach is when research explores areas where ‘we don't know that we don't know.’ This is the quadrant of true paradigm shifts arising from areas with low awareness and high innovation, and where the question ‘why do we fund this?’ finds its most satisfactory answer and leads to truly innovative and disruptive technologies.

Louis Pasteur famously said in an 1854 lecture regarding observation: ‘In the fields of observation chance favors only the prepared mind.’^5^ Breakthroughs and discoveries often occur to those who are educated, experienced, and actively looking for them. Biology provides many examples of such disruptive technologies arising from keen observations. For example, the discovery of penicillin emerged serendipitously.^6^ What began as an unexpected observation eventually transformed both medicine and microbiology. However, progress typically follows a slower and iterative process of discoveries leading to a gradual expansion of the knowledge boundary.

To understand how modern agriculture became what it is today, it is helpful to examine how the Green Revolution was achieved. The Green Revolution occurred over a relatively short period of time, from an anthropological perspective (Fig. 2(A)). The great increase in crop yields we are achieving today is not the result of a single inventive step but instead was achieved by incremental advances in our knowledge of mechanization, genetics and chemistry (Fig. 2(B)–(D)).

**Figure 2:**
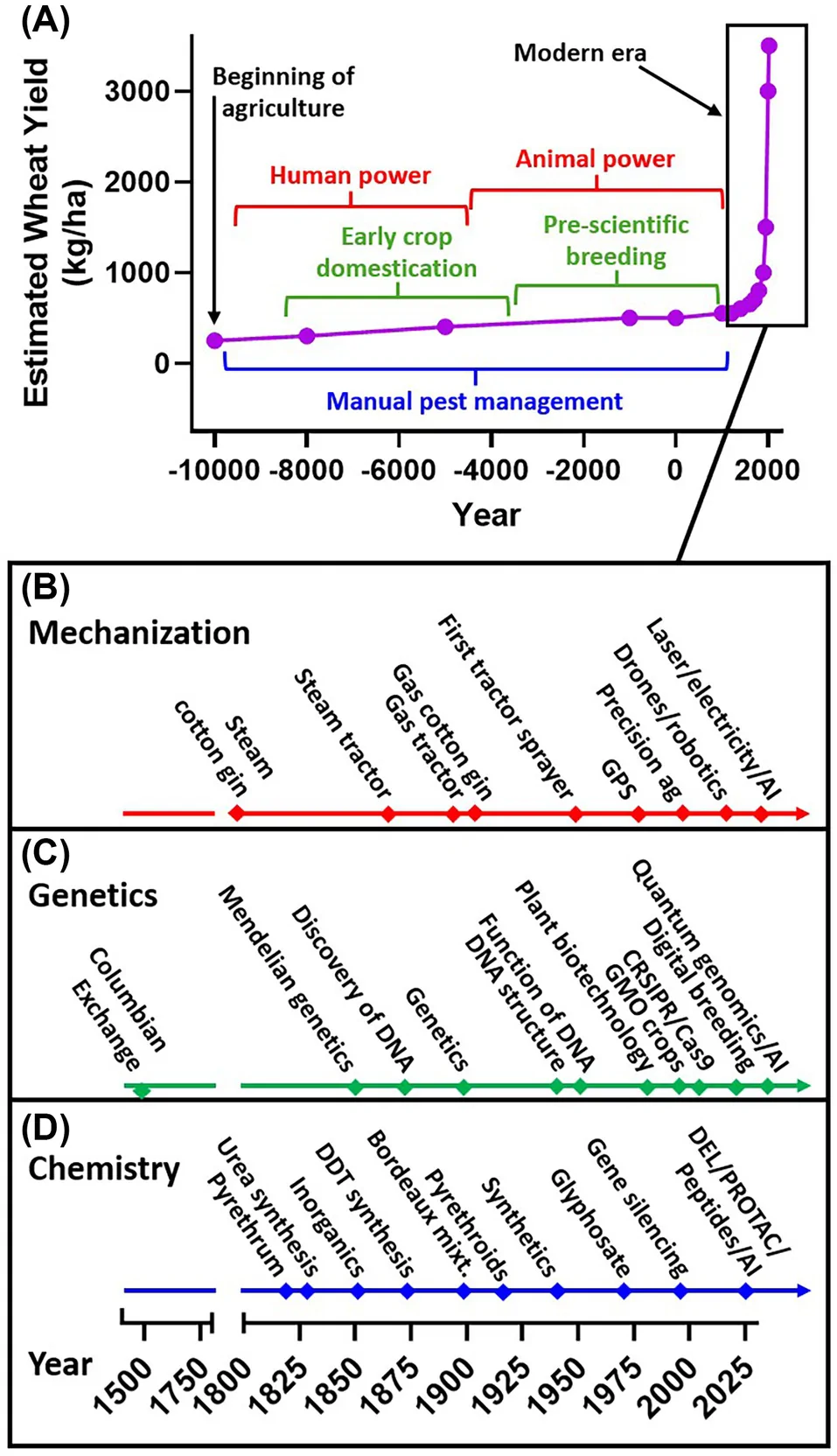
(A) Timeline of advances in agricultural practices leading to the Green Revolution using wheat yield as an example.^22^, ^23^ Technological advances in our knowledge of (B) mechanization (in red), (C) genetics (in green), and (D) chemistry (in blue), that enabled the Green Revolution and those that may impact future practices. AI = artificial intelligence, DEL = DNA‐encoded libraries, PROTAC = Proteolysis‐targeting chimera.

For most of humanity, crop yields remained low and relied initially on human power and then animal power until progressive advances in mechanization, starting with the steam engine cotton gin in 1750, to the introduction of gas‐powered farm equipment during the nineteenth and twentieth centuries (Fig. 2(B)).^7^ The introduction of machinery such as mechanical harvesters, tractors, and later precision‐guided equipment significantly altered the scale and efficiency of agricultural production. Mechanization reduced labor requirements, increased the speed of field operations, and enabled farmers to cultivate larger areas of land. At the same time, it introduced new research questions related to soil compaction, fuel efficiency, machinery design, and the integration of technology with biological systems. In modern agriculture, mechanization continues to evolve through automation, sensors, and precision agriculture technologies that allow farmers to manage crops and their pests more accurately.

Similarly, yields remained relatively low during the early crop domestication process and pre‐scientific breeding. Early plant breeding relied primarily on the selection of observable characteristics such as yield and resilience, without a clear grasp of the underlying genetic mechanisms at work. This began to change with the work of Gregor Mendel, whose principles of inheritance provided a theoretical foundation that transformed plant breeding into a more systematic and scientific practice.^8^ Subsequent discoveries, including the discovery of DNA and advances in molecular biology, genomics and bioinformatics, progressively expanded this framework, allowing the identification of specific gene and regulatory pathways associated with the desirable traits. The seemingly sudden sharp increase in yield during the Green Revolution is the result of a progressive increase in our knowledge (Fig. 2(C)).

Importantly, newer developments did not replace earlier approaches but refined and extended them. Traditional breeding methods remain essential, particularly for developing locally adapted crop varieties, while modern techniques such as marker‐assisted selection have increased precision and efficiency of selection processes.^9^ Contemporary innovations, including gene editing technologies, allow for the direct modification of plant genomes, introducing new possibilities (such as pest resilient crops) alongside uncertainties related to regulation, ecological interactions and long term stability.^10^

Finally, for most of human history, fertilization relied on manure amendment and pest management was achieved manually. However, the high yields achieved since the Green Revolution are the combined results of chemical fertilization and chemical pest management. Again, this occurred progressively. In fact, first organic molecule to be synthesized was urea, but its use as a fertilizer did not become important until the 1950s. Although the insecticidal activity of crushed *Tanacetum cinerariifolium* flowers was known in Persia as early as 400 bc for controlling household insects, the first chemical characterization of the active pyrethroids in pyrethrum was not achieved until 1922.^11^ The first synthetic chemical insecticide was DDT (1874), but its insecticidal activity was discovered 65 years later. While its use was banned in the United States in 1972 due to its persistence in the environment and toxicity to wildlife, its introduction marked a shift toward the use of synthetic organic chemistry in agriculture and numerous other pesticides followed. The next major innovation driven by our need to control insects required advances in plant biotechnology, with the release of genetically engineered insect‐resistant *Bacillus thuringiensis* (Bt) crops (starting in 1995) that express the cry gene from the soil bacterium *Bacillus thuringiensis*,^12^ illustrating that innovation does not occur in a vacuum, but rather often relies on concerted progress in multiple areas of science.

The first important step in chemical disease management occurred in 1882, with the Bordeaux mixture, a mix of copper sulfate and lime, was discovered accidentally in France to control downy mildew in vineyards (1882), and is considered the first effective agricultural fungicide, saving the European wine industry and becoming a staple for disease control in many crops.^13^ As with other pesticides, the next generations of fungicides started appearing in the 1940s. More recent advances in plant genetics have enabled breeding of crops with disease resistance traits, which is now coupled with genetic engineering and genome editing techniques, illustrating the dependence on cross‐disciplinary approaches driving scientific progress.^14^

Early weed control strategies relied primarily on mechanical cultivation and crop rotation.^15^ Similarly, the first selective herbicide (2,4‐D) was introduced in the mid‐1940s,^16^ more than 100 years after the first synthesis of urea (1828), followed by the rapid introduction of all the other chemical classes (Fig. 2(D)). Glyphosate, often considered a ‘once in a century herbicide,’ stands out as an excellent example of a serendipitous discovery. Monsanto Company was testing aminomethylphosphonic acid analogs as potential water‐softening agents and realized that a few of these molecules were herbicidal. Additional exploration of the chemical space surrounding these structures led to the discovery of the non‐selective herbicide glyphosate.^17^ Since its commercialization in 1974, it remains the only herbicide in its class and has become the most important herbicide in the world. An important aspect of its success is due to its use in conjunction with transgenic, glyphosate‐resistant crops, accounting for almost 90% of all transgenic crops grown worldwide.^18^

Initially, these technologies were viewed as highly effective solutions to weed pressure. However, the emergence of herbicide‐resistant weed populations revealed the limitations of that assumption. Species such as some of those of *Amaranthus* and *Lolium* evolved resistance to multiple herbicide modes of action, forcing researchers to reconsider earlier approaches.^19^, ^20^ What once appeared to be a stable point of knowledge instead exposed a new set of biological questions involving evolutionary selection, genetic variation, and ecological adaptation.^21^ This shift illustrates how new discoveries often reveal previously unrecognized complexity. Herbicides remain important tools, but their effectiveness now depends on integrating chemical control with cultural and ecological management practices.

This retrospective view of pest management in agriculture highlights that most of the progress has been achieved incrementally, mostly by researchers expanding the boundary of what is known by exploring knowledge gaps. However, researchers occasionally stumbled in unknown areas resulting in truly innovative discoveries. It should be noted that using this time scale, crop yields are increasing exponentially, but recent data suggest that this trend is plateauing because conventional breeding is reaching the crop biological limits in photosynthesis and harvest index.^24^ Furthermore, climate change, soil degradation, water scarcity, and pest resistance impose additional strains on crop productivity. Future increases will likely rely on gene editing crop improvement, and precision new technological innovations in equipment and pest management tools.

Looking forward, what future technologies will drive the next phase of agricultural innovation? Undoubtedly, the adoption of artificial intelligence (AI) in all aspects of science will accelerate the expansion of the knowledge boundary, assuming that the proper large language models are developed. As mechanization generates high‐resolution spatial and temporal datasets, genetic technologies produce increasingly detailed genomic information, and chemical innovations introduce more targeted modes of action, the limiting factor shifts from data acquisition to data synthesis and decision making.

Machine learning approaches are particularly effective in pattern recognition, predictive analytics, and the identification of relationships within large multidimensional datasets. These capabilities are expected to support predictive management strategies by linking environmental signals, crop responses, and intervention outcomes, thereby improving agricultural efficiency and precision. However, despite rapid advances in AI‐assisted analytics, current models remain constrained by their dependence on historical training data and limited capacity for mechanistic reasoning and causal inference. In biological systems, where environmental variability and emergent interactions frequently exceed the bounds of existing datasets, unsupervised extrapolation may generate biologically implausible or unsupported conclusions. Consequently, AI should be regarded as a decision support tool that augments scientific interpretation rather than a substitute for human expertise and experimental validation.^25^

Advances in quantum computing are likely to further expand computational capacity by enabling the rapid solution of high‐dimensional optimization and simulation problems in genomics and protein identification,^26^ as well as improving the resolution of their functional roles. Computational advances not only improve visualization of processes within pest organisms but also enhance the discovery of new pesticidal compounds through predictive modeling and mechanistic simulation.^27^

The trajectory of agricultural mechanization suggests a transition toward highly autonomous, sensor‐integrated production systems. Robotics and drone platforms will become standard components of field operations, enabling continuous, detailed mapping, monitoring and precise intervention against pest organisms. Hyperspectral imaging and other sensing technologies are expected to improve diagnostic accuracy for nutrients, disease, and pest pressures. While laser and electrical weed control systems are slated to reduce dependence on chemical herbicides. The convergence of these tools points toward a production model defined by precision, reduced labor intensity, and lower environmental susceptibility.

In the domain of genetics, gene editing technologies, such as CRISPR/Cas9 and others, are positioned to substantially accelerate the development of crop varieties adapted to climatic variability and evolving biotic stressors. The capacity to directly modify target genomic sequences without adding foreign genetic material, enables targeted activation of traits that would be difficult to achieve through conventional breeding alone. At the same time, editing crop plants to incorporate competitive or ‘weediness’ characteristics and insect or disease resistance traits may offer new pathways for ecological pest management. Furthermore, gene‐drives, which promote the preferential inheritance of specific genetic traits, are being explored and trialed to control certain species of pest insects, such as mosquitoes carrying malaria.

Chemical innovation is moving further toward specificity and biological integration, with emerging modalities such as peptide‐based pesticides, RNA interference (RNAi), and protein targeted degradation strategies offering new mechanisms for crop protection. These approaches have the potential to reduce non‐target effects and slow the development of resistance relative to currently available conventional chemistries.

The growth of scientific knowledge therefore resembles the expansion of a constellation rather than the illumination of a single path. Each new insight adds another point of light, but its intensity depends on how it connects with others. Strengthening these connections may ultimately be as important as the discoveries themselves. By preserving, sharing, and integrating knowledge across disciplines, the knowledge boundary can continue to expand, enabling agriculture to respond more effectively to an ever more uncertain future.

## Data Availability

This is a perspective and we do not present scientific data. Just an opinion on a topic.
